# JNK suppresses melanogenesis by interfering with CREB-regulated transcription coactivator 3-dependent MITF expression

**DOI:** 10.7150/thno.41502

**Published:** 2020-03-04

**Authors:** Ji-Hye Kim, A-reum Hong, Yo-Han Kim, Hanju Yoo, Sang-Wook Kang, Sung Eun Chang, Youngsup Song

**Affiliations:** 1 Department of Biomedical Sciences, University of Ulsan College of Medicine, Seoul 05505, Korea.; 2 Bio-Medical Institute of Technology (BMIT), University of Ulsan College of Medicine, Seoul 05505, Korea.; 3 Department of Dermatology, University of Ulsan College of Medicine, Seoul 05505 Korea.; 4 Asan Institute for Life Sciences, Asan Medical Center, Seoul 05505, Korea.

**Keywords:** CREB regulated transcription coactivator 3 (CRTC3), CREB, melanogenesis, Ro31-8220, JNK

## Abstract

Melanogenesis is a critical self-defense mechanism against ultraviolet radiation (UVR)-induced skin damage and carcinogenesis; however, dysregulation of melanin production and distribution causes skin-disfiguring pigmentary disorders. Melanogenesis is initiated by UVR-induced cAMP generation and ensuing activation of transcription factor CREB, which induces expression of the master melanogenic regulator MITF. Recent studies have demonstrated that recruitment of CRTCs to the CREB transcription complex is also required for UVR-stimulated melanogenesis. Therefore, modulation of cAMP-CRTC/CREB-MITF signaling may be a useful therapeutic strategy for UVR-associated skin pigmentary disorders.

**Methods**: We identified the small-molecule Ro31-8220 from CREB/CRTC activity screening and examined its melanogenic activity in cultured mouse and human melanocytes as well as in human skin. Molecular mechanisms were deciphered by immunoblotting, RT-PCR, promoter assays, tyrosinase activity assays, immunofluorescent examination of CRTC3 subcellular localization, and shRNA-based knockdown.

**Results**: Ro31-8220 suppressed basal and cAMP-stimulated melanin production in melanocytes and human melanocyte co-culture as well as UVR-stimulated melanin accumulation in human skin through downregulation of MITF and tyrosinase expression. Mechanistically, down regulation of MITF expression by Ro31-8220 was due to inhibition of transcriptional activity of CREB, which was resulted from phosphorylation-dependent blockade of nuclear translocation of CRTC3 via JNK activation. The selective JNK activator anisomycin also inhibited melanin production through phosphoinhibition of CRTC3, while JNK inhibition enhanced melanogenesis by stimulating CRTC3 dephosphorylation and nuclear migration.

**Conclusions**: Melanogenesis can be enhanced or suppressed via pharmacological modulation of a previously unidentified JNK-CRTC/CREB-MITF signaling axis. As Ro31-8220 potently inhibits UVR-stimulated melanin accumulation in human skin, suggesting that small-molecule JNK-CRTC signaling modulators may provide therapeutic benefit for pigmentation disorders.

## Introduction

The primary function of skin tissue is to provide first-line protection against external hazards, which makes it the most vulnerable tissue to these threats. For instance, skin tissue is directly exposed to ultraviolet radiation (UVR), which renders skin tissue highly susceptible to carcinogenesis via somatic mutations through the generation of reactive oxygen and nitrogen species 1. As a self-defense mechanism, humans have evolved a skin pigmentary system. Melanin is a heterogeneous mixture derived through the polymerization and oxidation of phenols (tyrosine), and its special chemical structure confers skin color and photoprotection by absorbing UV and reactive species 2. In human skin, melanogenesis is tightly controlled by microphthalmia-associated transcription factor (MITF), the master regulator of pigment gene expression. Various extracellular signals are transduced via intracellular signaling pathways that converge on MITF. Thus, MITF integrates upstream signaling pathways to regulate the downstream pigmentation genes tyrosinase (Tyr), tyrosinase related protein-1 (TYRP-1), and dopachrome tautomerase (DCT). Tyrosinase, the rate-limiting enzyme in melanin biosynthesis, controls melanogenesis by catalyzing the hydroxylation of L-tyrosine to L-dihydroxyphenylalnine (L-DOPA) as well as the oxidation of DOPA to DOPA-quinone. The most available topicals for the treatment of skin hyperpigmentary disorders are tyrosinase inhibitors; in that context, multiple agents have been developed focusing on the mechanism of enzyme tyrosinase inhibition.

The most physiologically significant melanogenic stimulus is UVR. Exposure to UV successively activates cyclic AMP (cAMP) production, cAMP-dependent kinase PKA, and the transcription factor cAMP response element-binding protein (CREB), which in turn induces the expression of MITF and downstream target melanogenic genes. In addition to CREB phosphorylation by PKA, recent studies have demonstrated that the recruitment of CREB-regulated transcription coactivator (CRTC)3 to the CREB transcription complex is also required for cAMP-stimulated MITF expression and melanogenesis 3.

While melanogenesis is critical for protection of skin from UVR-induced damage, excessive or uneven deposition can cause skin-disfiguring morbidities with potential psychosocial impact. Moreover, excessive epidermal melanin may enhance the production of singlet O_2_, causing DNA damage 4. Both hyperpigmentary conditions such as uneven skin tone, mottling, solar lentigo, melasma, and post-inflammatory hyperpigmentation (PIH) and hypopigmentation disorders such as post- inflammatory hypopigmentation, vitiligo, and contact leukoderma occur very frequently across ethnicities. To reverse the abnormal production of melanin or melanocytic survival in pigmentary disorders, researchers have endeavored to identify substances that can effectively regulate melanogenesis. However, aside from tyrosinase activity modulators, few compounds with the requisite safety and efficacy are available. Given the importance of the cAMP-PKA-CRTC/CREB-MITF pathway in melanogenesis 3, 5, 6, we speculated that chemicals or agents that block or activate this axis may have anti- or pro-melanogenic activity, respectively, and thus provide potential therapeutic agents for pigmentary disorders. In this study, we identified Ro31-8220 as a potential inhibitor of CRTC/CREB-MITF signaling and characterized both its anti-melanogenic potential and molecular basis of action via JNK activation and ensuing inhibitory phosphorylation of CRTC3. In addition, we demonstrate that JNK inhibition enhances MITF expression via this pathway. Thus, bidirectional regulation of JNK-CRTC/CREB-MITF signaling may be an effective therapeutic strategy against both hypo- and hyperpigmentation disorders.

## Materials and Methods

### Chemicals

The SCREEN-WELL Kinase Inhibitor library (Enzo Life Sciences, New York, NY, USA) and Clinical Compound Library (TargetMol, Wellesley Hills, MA, USA) were used for screening for CREB activity. Ro31-8220 (3-[3-[2,5-Dihydro-4-(1-methyl-1*H*-indol-3- yl)-2,5-dioxo-1*H*-pyrrol-3-yl]-1*H*-indol-1-yl]propyl carbamimidothioic acid ester mesylate), forskolin (FSK,[3*R*-(3α,4aβ,5β,6β,6aα,10α,10aβ,10bα)]-5-(Acetyloxy)-3-ethenyldodecahydro-6,10,10b-trihydroxy-3,4a,7,7,10a-pentamethyl-1*H*-naphtho[2,1-*b*]pyran-1-one), anisomycin ((2*R*,3*S*,4*S*)-2-[(4-Methoxyphenyl)methyl]-3,4-pyrrolidinediol 3-acetate), and SP600125 (Anthra[1-9-*cd*]pyrazol-6(2*H*)-one) were purchased from Tocris Bioscience (Bristol, UK). Unless specified, Ro31-8220 was applied at 1-μM, FSK at 10 μM, anisomycin at 30 nM, and SP600125 at 15 μM.

### Cell culture

Mouse embryonic fibroblasts (MEFs), 293T cells, and B16F10 melanoma cells were maintained in Dulbecco's modified Eagle's medium (DMEM) supplemented with 10% fetal bovine serum (FBS) (Corning Life Sciences, Corning, NY, USA) and 1% penicillin/streptomycin. Mel-Ab mouse melanocytes were cultured in DMEM supplemented with 10% FBS, 100 nM 12-O-tetradecanoylphorbol-13-acetate (Sigma-Aldrich, St. Louis, MO, USA), 1 nM cholera toxin (Cayman Chemicals, Ann Arbor, MI, USA), and 1% penicillin/streptomycin. shRNAi-based CRTC3 knockdown Mel-Ab cells were prepared and maintained as described previously 7. Primary normal human melanocytes obtained from Invitrogen (Carlsbad, CA, USA) were maintained in Medium 254 (Thermo Fisher, Waltham, MA, USA) supplemented with Human Melanocyte Growth Supplement (Thermo Fisher) and primary human keratinocytes in Cascade Biologics EpiLife (Thermo Fisher) supplemented with Human Keratinocyte Growth Supplement (Thermo Fisher). For co-culture experiments, primary human melanocytes were first seeded on 6-well plates at 6 × 10^4^ cells/well, followed the next day by seeding of primary human keratinocytes at 3 × 10^5^ cells/well (for a melanocyte to keratinocyte ratio of 1:5) with 1.5 nM ET-1 (R&D Systems, Minneapolis, MN, USA) and 50 ng/mL SCF (Sigma-Aldrich).

### Transcriptional activity of CREB/CRTC3

The transcriptional activity of CREB/CRTC3 was assessed using a promoter-based luciferase reporter assay. Briefly, the EVX1 (220 bp), NR4a2 (664 bp), Rgs2 (1000 bp) 8, MITF (494 bp), or Tyr (390 bp) promoters cloned separately into pGL3 plasmids were transfected into HEK-293T cells using polyethylenimine (PEI) reagents. After 24 h of transfection, cells were treated as indicated in the figures for 6 h and firefly luciferase activity measured.

### Melanin content and tyrosinase activity assay

Mel-Ab cells in DMEM supplemented with 10% FBS and 1% penicillin/streptomycin were treated with FSK, Ro31-8220, anisomycin, and (or) specific vehicles as indicated in the figures. In all cases, pretreatment with Ro31-8220 or anisomycin started 30 min prior to FSK. The culture medium containing vehicle or drugs was replaced every 24 h. At 96 h of treatment, cells were solubilized with 1 N NaOH, boiled for 30 min with intermittent vortexing, and centrifuged. Melanin in the supernatant was measured as the optical density at 405 nm using a microplate reader (Biotek, Winooski, VT, USA). Melanin content was normalized to lysate total protein and is expressed as percent change relative to vehicle-treated controls. Tyrosinase activity was measured as described previously 9. Tyrosinase activity was also normalized to lysate total protein and expressed as percent change relative to the vehicle-treated controls.

### Cell viability assay

Cell viability was assessed using the MTT assay (Duchefa-biochemie, Haarlem, Netherlands) following the manufacturer's instructions. Results are reported as the percent change relative to vehicle-treated controls.

### Antibodies and immunoblotting

Protein samples from cultured cells were prepared in lysis buffer containing 10 mM Tris, 5 mM EDTA, and 1% SDS (pH 7.4), separated by 5.5%-10% SDS PAGE, and transferred to nitrocellulose membranes (ATTO Technology, Amherst, NY, USA). Membranes were blocked with 3% BSA (Bovogen Biologicals, Keilor East, Australia) in TTBS, and subjected to immunoblotting. Protein samples from human skin tissues were prepared by first grinding the tissue in liquid nitrogen prior to lysis in buffer containing 20 mM HEPES, 150 mM NaCl, 1 mM EDTA, 1 mM EGTA, 1% Triton X-100, protease inhibitor cocktail (Tech & Innovation, ChunCheon, Korea), and phosphatase inhibitors (5 mM Na-pyrophosphate, 20 mM β-glycerophosphate, and 50 mM NaF). Antibodies against ACC, phospho-ACC, Raptor, phospho-Raptor, AMPK, phospho-AMPK, JNK, and phospho-JNK were purchased from Cell Signaling Technology (Danvers, MA, USA); tyrosinase, TYRP-1, and DCT antibodies from Santa Cruz Biotechnology (Dallas, TX, USA); and MITF antibody from Neomarkers (Fremont, CA, USA). The CRTC3 antibody was produced in-house and CRTC2, CREB, and phospho-CREB antibodies were acquired from the Montminy laboratory (Salk Institute for Biological Studies, La Jolla, CA, USA). GAPDH (Thermo Fisher Scientific, Waltham, MA, USA) and α-tubulin (Merck Millipore, Burlington, MA, USA) were used as internal loading controls.

### Gene expression analysis by qRT-PCR

Total RNA was isolated using the FavorPrep Blood/Cultured Cell Total RNA purification Kit (Farvorgen Biotech, Changzhi Township, Taiwan). A 700-ng sample was used for first strand cDNA synthesis with the ReverTra Ace qPCR RT Kit (Toyobo, Osaka, Japan) and random hexamer according to the manufacturer's instructions. The relative expression levels of target mRNAs were compared by quantitative real-time reverse transcriptase-PCR (qRT-PCR) using the THUNDERBIRD SYBR qPCR mix (Toyobo) and Lightcycler 480 (Roche Applied Science, Indianapolis, IN, USA). Expression of L32 was used as an internal reference. Specific primer sets for amplifying target genes are listed in Table S1.

### Subcellular localization of CRTC3

Plasmid constructs encoding the CRTC3-EGFP fusion gene were transfected into B16F10 cells using PEI reagents. After 24-48 h of transfection, CRTC3-EGFP-transfected B16F10 cells were treated with vehicle, Ro31-8220, FSK, anisomycin, and (or) SP600125 as indicated in the figures and subcellular localization of CRTC3 was monitored by fluorescence microscopy (TS100, Nikon Instech, Tokyo, Japan).

### JNK induced CRTC3 phosphorylation

Plasmid constructs encoding the CRTC3 and JNK1 (Addgene, Watertown, MA, USA, cat#: 19726), JNK2 (Addgene, cat#: 19727), or JNK3 (addgene, cat#: 19729) were transfected into HEK-293T cells using PEI reagents; 24 h after transfection, protein samples were prepared in lysis buffer containing 10-mM Tris, 5-mM EDTA, and 1% SDS (pH 7.4) and separated by 5.5% SDS-PAGE and subjected to immunoblotting.

### *Ex vivo* human skin cultures

Skin tissue was acquired from patients receiving neck or abdomen reduction surgery with informed consent in accordance with IRB number 2014-0837. Skin tissues were briefly washed with 100% EtOH followed by 70% EtOH, cut into approximately 1 cm^2^ sections and placed on metal grids in 6-well plates in contact with DMEM containing 5% FBS and 5% penicillin/streptomycin plus either vehicle or Ro31-8220 under a humidified environment of 5% CO_2_. Culture medium containing vehicle or drugs was replaced every day. For UVR-stimulated melanogenesis, skin tissue was exposed to 150 mJ UVB for 50 s. After 96 h, skin tissues were harvested and divided into two pieces. One part was embedded in paraffin for histology and immunohistochemistry and the other ground in liquid nitrogen for isolation of protein samples as described above.

### Fontana-Masson staining, melanin index, and immunohistochemistry

Paraffin-embedded human skin tissues were cut into 5-μm thick sections and subjected to Fontana-Masson staining (ScyTek Laboratories, Logan, Utah, USA) according to the manufacturer's instructions. Multiple areas were randomly photographed using a phase-contrast microscope (BX53, Olympus, Tokyo, Japan) and the melanin index was determined by measuring the stained area normalized to total epidermal area using Image J (National Institute of Health, Bethesda, MA, USA) and expressed as percent change relative to vehicle-treated controls. For immunohistochemistry, paraffin-embedded 5-μm thick human skin sections were mounted on slides and rehydrated in descending concentrations of EtOH. After washing with TBST, antigen was retrieved by incubating slides in a streamer containing 10 mM citrate buffer for 20 min. Slides were then incubated in 3% H_2_O_2_ for 10 min, followed by 3N HCl for 30 min. After washing with TBST, slides were incubated with 3% BSA in TBST for 1 h and then with primary antibodies. Immunostaining was visualized by Fast-red staining.

### Statistical analysis

All data are expressed as mean ± standard error of the mean (s.e.m). Treatment group means were compared by unpaired Student's t-test using GraphPad Prism program. p < 0.5, p < 0.1, p < 0.01 represent *, **, and *** respectively (two-tailed) and were considered significant for all tests.

## Results

### Ro31-8220 inhibits CREB-mediated melanogenic gene transcription and suppresses melanin accumulation

**Figure 1:**
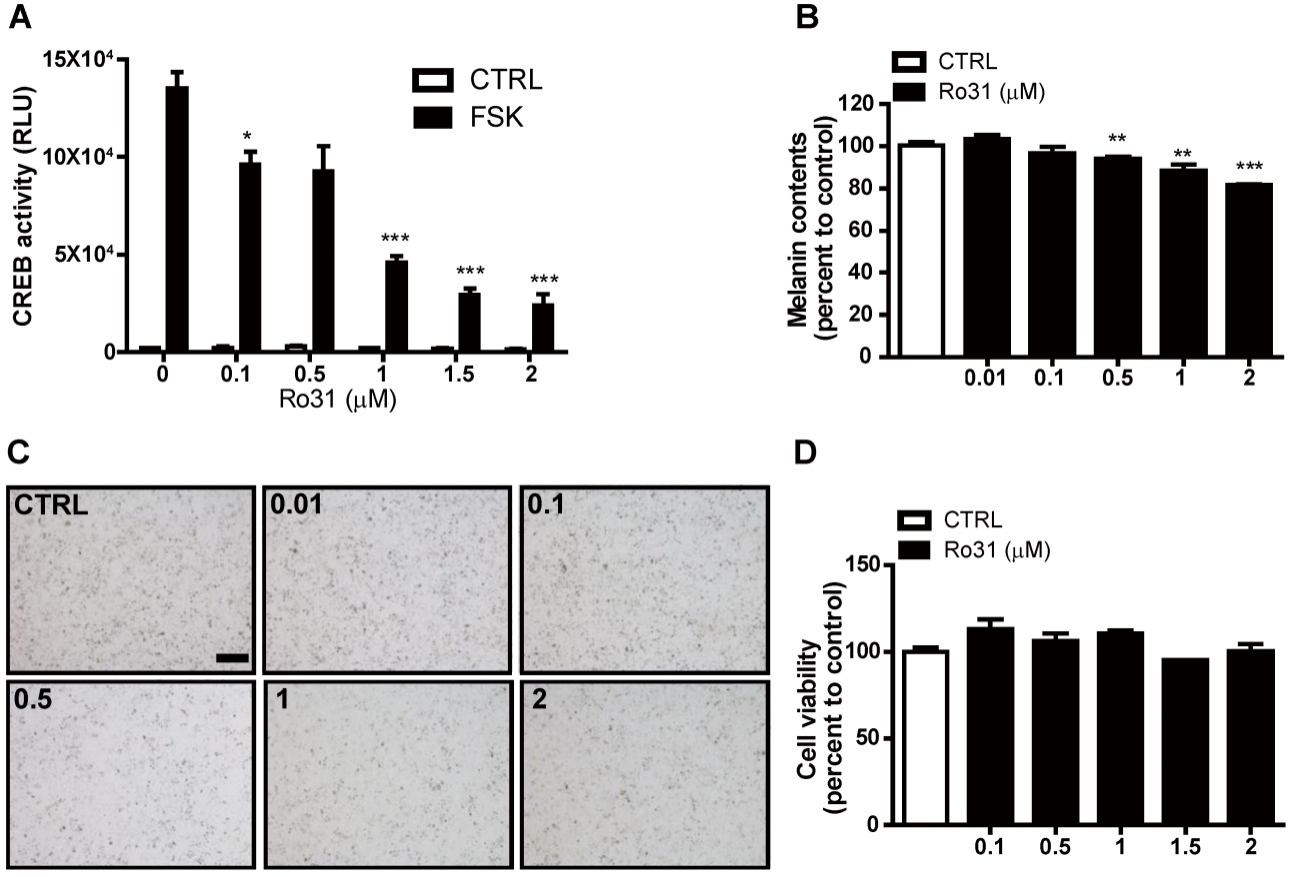
** Suppression of cAMP-stimulated CREB activity and melanin production by Ro31-8220.** (**A**) Ro31-8220 (0.1-2 µM) suppressed forskolin (FSK)-stimulated CREB activity as measured by hEVX1 promoter activity. (**B**, **C**) Melanin content of Mel-Ab cells was dose-dependently reduced by Ro31-8220 treatment for 96 h. Bars = 2000 µm. (**D**) Mel-Ab cell viability was not substantially reduced following treatment with 0.1-2 µM Ro31-8220 for 96 h according to MTT assay. CTRL: vehicle-treated controls, FSK: forskolin, Ro31: Ro31-8220, number in (**C**) indicates concentration (µM) of Ro31-8220.

To identify potential chemical melanogenesis modulators, we conducted a small-molecule drug screen by measuring transcription of human EVX1, a highly cAMP-sensitive and CREB-dependent gene, using a promoter-based CREB reporter assay system 7. This screen identified Ro31-8220 as a candidate small molecule with inhibitory activity against CREB-dependent transcription. At 0.1 µM, Ro31-8220 decreased EVX1 promoter activity by 30% in response the PKA activator forskolin (FSK), and higher Ro31-8220 doses further suppressed EVX1 promoter activity (Figure 1A). We then examined if Ro31-8220 reduces MITF expression and exhibits anti-melanogenic activity in cultured melanocytes. Indeed, Mel-Ab cells treated with 0.1 µM Ro31-8220 accumulated 4% less melanin, while 1 µM and 2 µM Ro31-8220 treatments reduced melanin content by 12% and 19%, respectively, compared with vehicle-treated controls (Figure 1B, C). Moreover, these concentrations of Ro31-8220 did not alter Mel-Ab cell viability, suggesting that the anti-melanogenic activity did not result from nonspecific cytotoxicity (Figure 1D).

**Figure 2:**
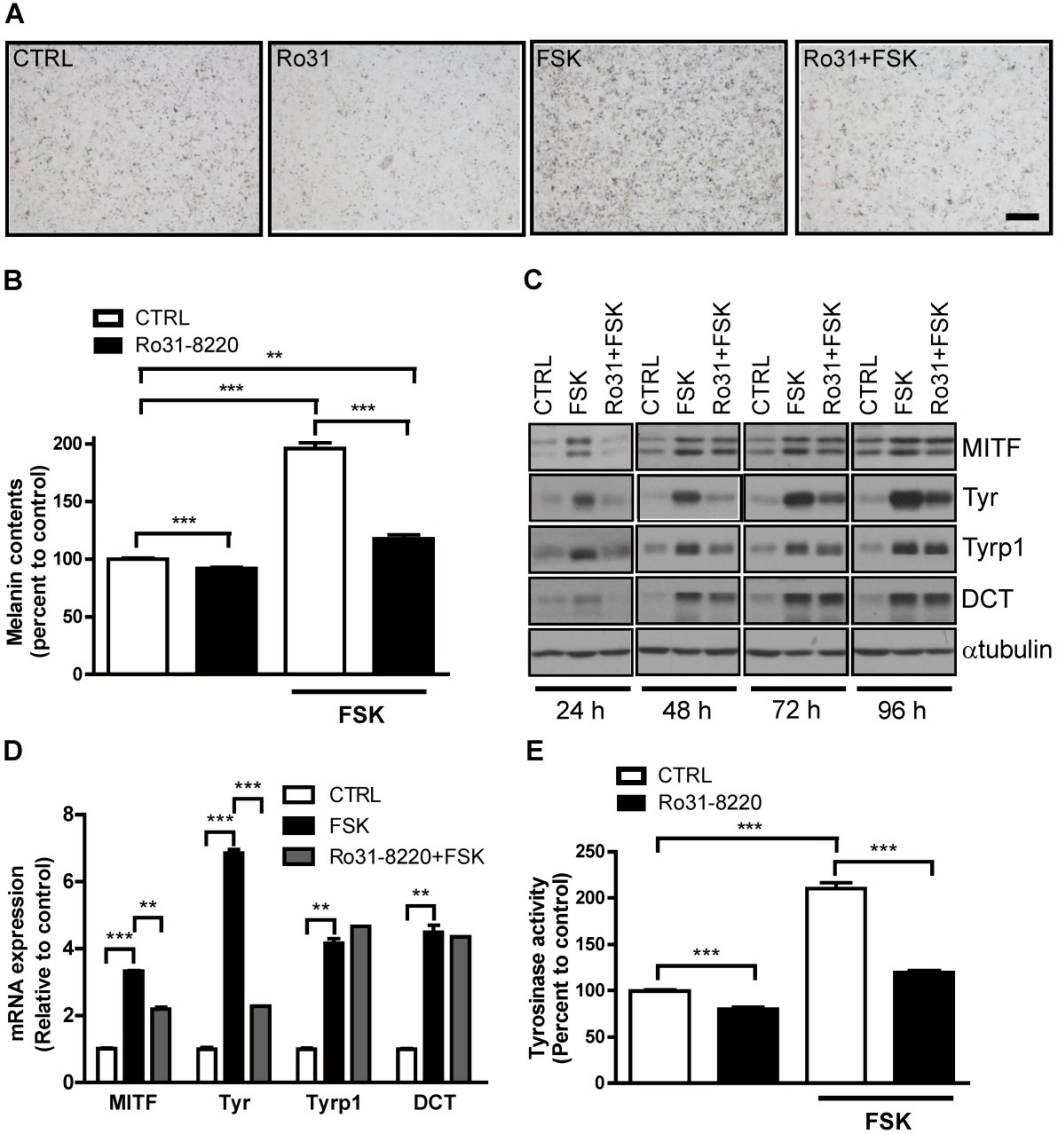
** Ro31-8220 downregulates cAMP-stimulated melanin accumulation and melanogenic gene expression in melanocytes.** Ro31-8220 suppressed FSK-induced induction of melanin and melanogenic genes. (**A**, **B**) Basal and FSK-stimulated melanin content of Mel-Ab cells after 96 h of vehicle or Ro31-8220 treatment. Bars = 2000 µm. (**C**, **D**) Expression levels of melanogenic genes at the protein level (**C**) and mRNA level (**D**) in Mel-Ab cells treated with vehicle (CTRL), FSK, or Ro31-8220 plus FSK (Rot + FSK) as revealed by western blotting and qRT-PCR, respectively. Ro31-8220 suppressed FSK-induced upregulation of tyrosinase activity. (**E**) Tyrosinase activity of Mel-Ab cells after treatment with vehicle (CTRL), Ro31-8220 (Ro31), FSK, or Ro31-8220 + FSK for 96 h (expressed as percent change from vehicle-treated control).

Physiological melanogenesis induction by UVR exposure involves stimulation of α-MSH secretion from keratinocytes, α-MSH binding to MC1R on melanocytes, and triggering of the cAMP-PKA-CREB signaling cascade 10. As expected, FSK enhanced melanin production in Mel-Ab cells, and this response was almost completely attenuated by pretreatment with Ro31-8220 (Figure 2A, B). Next, we examined whether this reduced melanin accumulation was associated with altered melanogenic gene expression. Indeed, the expression levels of the melanogenic genes MITF and tyrosinase were markedly elevated in Mel-Ab cells by FSK treatment, while pretreatment with Ro31-8220 inhibited melanogenic gene induction (Figure 2C, D). In addition, the elevation in tyrosinase activity induced by FSK in Mel-Ab cells was substantially reduced by Ro31-8220 pretreatment (Figure 2E). In contrast, treatment with Ro31-8220 alone for 2 h did not alter tyrosinase activity (Figure S1), indicating that Ro31-8220 does not directly inhibit tyrosinase catalytic activity.

### Ro31-8220 suppresses MITF expression and nuclear translocation of CRTC3

**Figure 3:**
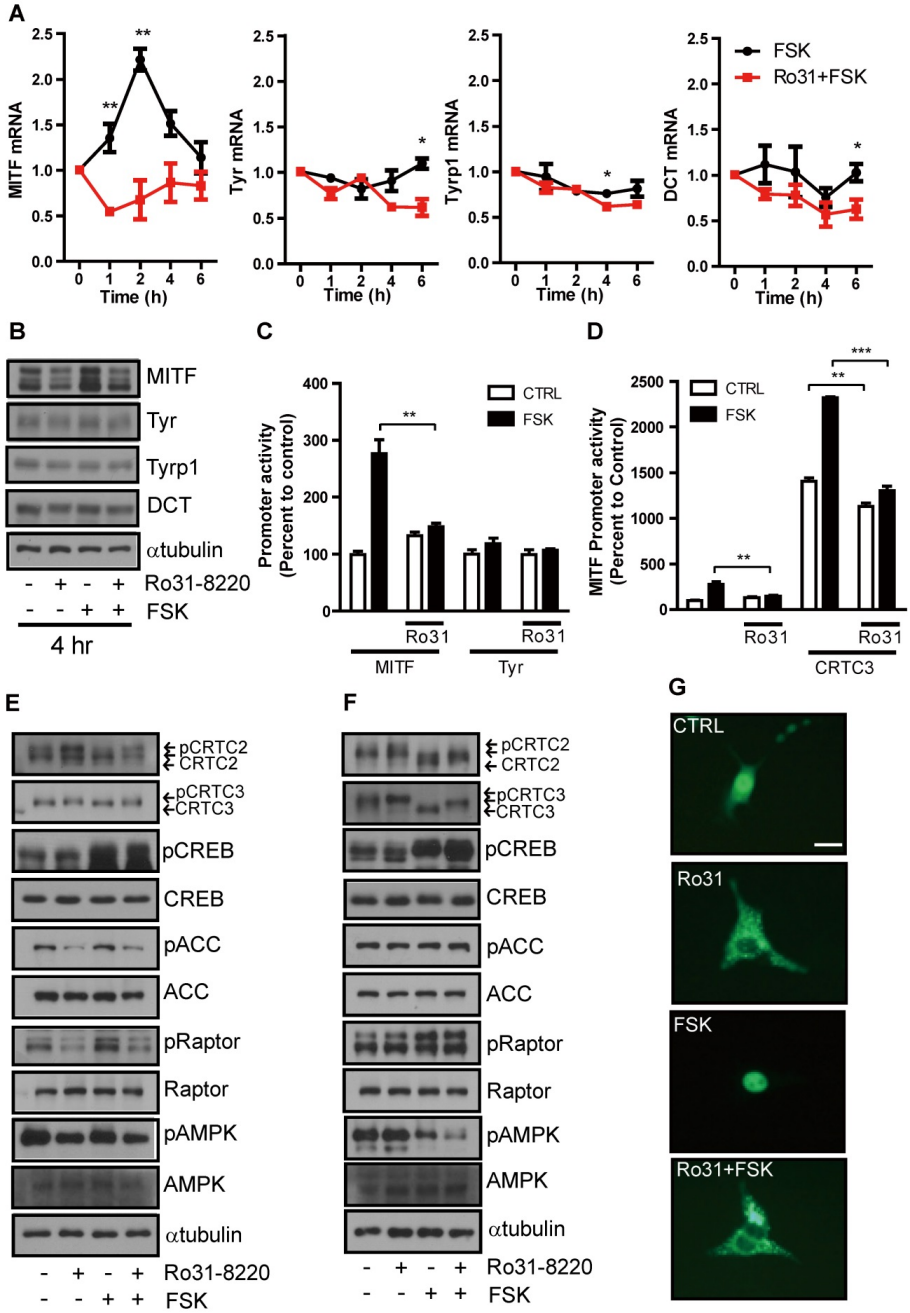
** Ro31-8220 suppresses MITF expression through phosphorylation-dependent inhibition of CRTC3 nuclear translocation.** (**A**) Short-term effects of FSK, and Ro31-8220 (Ro31) + FSK treatment (1-6 h) on expression levels of melanogenic gene mRNAs. (**B**) Short-term effects Ro31, FSK, and Ro31 + FSK treatment (4 h) on expression of melanogenic proteins. (**C**) The effect of Ro31-8220 (Ro31) on MITF and tyrosinase transcription levels using MITF and Tyr promoter-luc-based promoter reporter assays, respectively. (**D**) Effect of Ro31 on FSK- and CRTC3-stimulated MITF promoter activity assessed using a MITF-luc promoter reporter assay. (**E**, **F**) Expression levels and phosphorylation status of CRTC2/3, CREB, and AMPK signaling pathway proteins (AMPK, Raptor, ACC) analyzed by immunoblotting in Mel-Ab cells (**E**) and mouse embryonic fibroblasts (MEFs) (**F**) following 1 h of FSK treatment with or without 1 h of Ro31-8220 pretreatment. (**G**) Effects of Ro31-8220, FSK, and both on the subcellular localization of CRTC3 in B16F10 cells transfected with CRTC3-EGFP. Bar = 100 µm.

These inhibitory effects on cAMP-induced tyrosinase expression and melanin production suggest that Ro31-8220 may inhibit CREB and ensuing MITF expression. The mRNA and protein expression levels of NR4A2, a well-known CREB target gene (Figure S2A), and MITF in Mel-Ab cells are significantly increased at 1-2 h and 4 h, respectively, by FSK treatment (Figure 3A, B). This relatively rapid rise was followed by a decline, consistent with the well-known burst-attenuation kinetics of CREB transcriptional target gene expression. FSK-stimulated MITF expression was strongly attenuated by Ro31-8220 pretreatment (Figure 3A, B), while short-term treatment with FSK or Ro31-8220 did not alter mRNA and protein expression of tyrosinase, TYRP-1, and DCT (Figure 3A, B), arguing against nonspecific effects on MITF expression. Moreover, while Ro31-8220 inhibited FSK-stimulated MITF, NR4a2, Rgs2 promoter, and CREB activity, it had no effect on tyrosinase promoter activity (Figure 3C, D and Figure S2B-D).

To elucidate the molecular mechanism underlying inhibition of CREB transcriptional activity by Ro31-8220, we first examined the phosphorylation status of CREB and found that neither basal nor FSK-induced CREB phosphorylation level was reduced by Ro31-8220 (Figure 3E, F). An AMPK-mediated phosphorylation-dependent CRTC3 interaction with 14-3-3 protein is known to inhibit CRTC nuclear translocation and cAMP-dependent CREB target gene expression 11, 12, so we investigated whether this pathway is involved in CREB transcriptional activity suppression by Ro31-8220. Indeed, CRTC2/3 phosphorylation was elevated in Ro31-8220-treated Mel-Ab cells and mouse embryonic fibroblasts (MEFs), and Ro31-8220 treatment attenuated FSK-stimulated dephosphorylation of CRTC2/3 compared with vehicle-treated controls (Fig. 3E, F). Consistent with increased phosphorylation, Ro31-8220 treatment also promoted cytoplasmic localization of CRTC3 and inhibited FSK-stimulated nuclear migration of CRTC3 (Figure 3G). Unexpectedly, the increase in CRTC phosphorylation by Ro31-8220 was not related to AMPK activity as Ro31-8220 had no significant effect on AMPK expression and did not stimulate AMPK or its upstream signals, such as calcium flux and mitochondrial membrane potential, which regulate AMPK activity (Figure 3E, F and Figure S3). These findings imply that phosphorylation of CRTC by Ro31-8220 involves a signaling pathway other than AMPK.

### JNK suppresses melanogenesis by promoting phosphorylation and CRTC cytoplasmic retention

**Figure 4:**
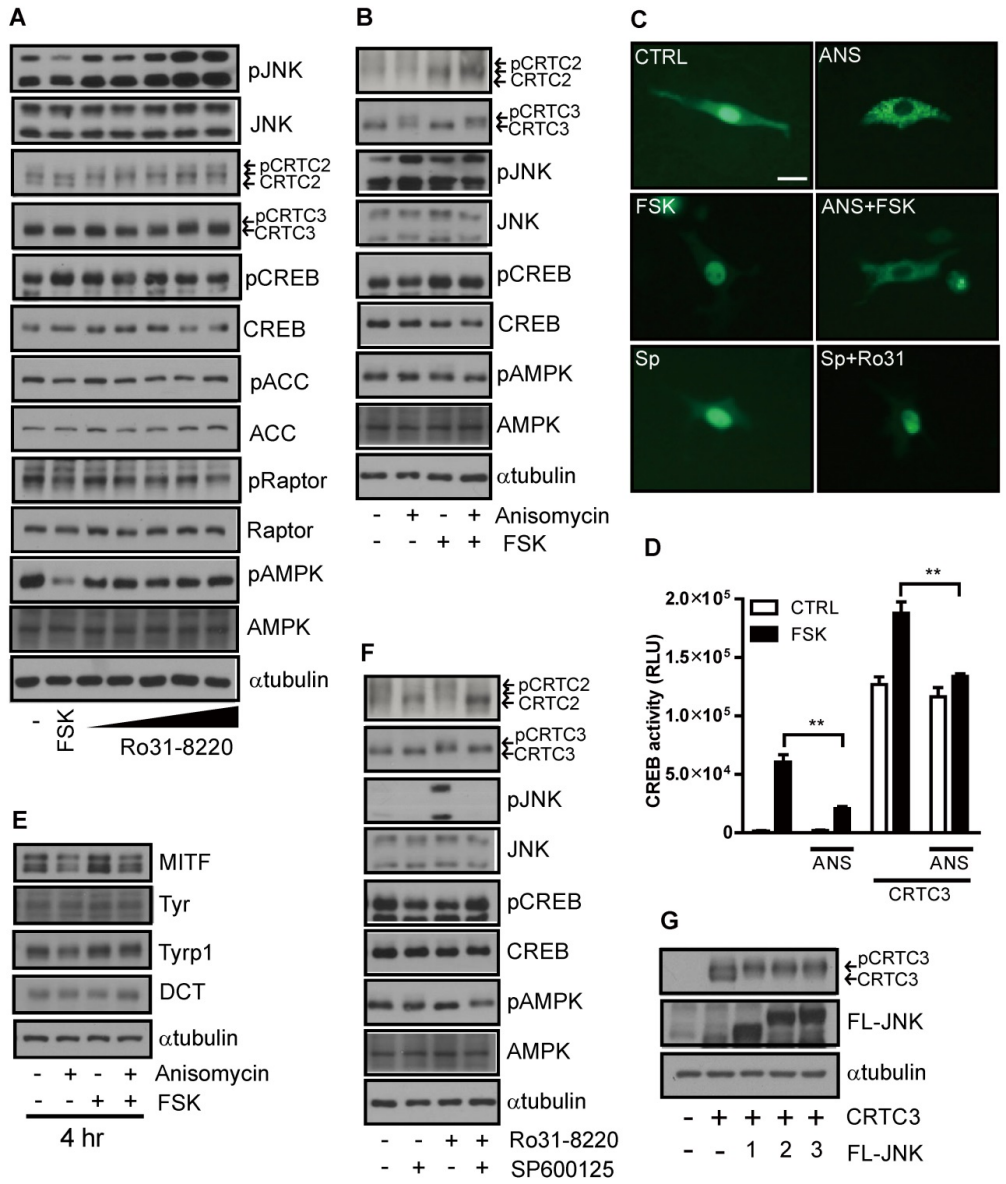
** JNK mediates Ro31-8220-induced suppression of melanogenesis through inhibitory phosphorylation of CRTC.** (**A**) Mel-Ab cells were treated with FSK or Ro31-8220 for 1 h and the expression and phosphorylation levels of CRTC2/3, CREB, AMPK, and JNK were examined by western blotting. (**B**) Mel-Ab cells were treated with anisomycin, FSK, or anisomycin for 1 h followed by FSK for 1 h, and the phosphorylation and expression level of CRTC2/3, CREB, AMPK, and JNK were analyzed by western blotting. (**C**) B16F10 cells transfected with CRTC3-EGFP were treated with anisomycin (ANS), FSK, anisomycin plus FSK, SP600125 (SP), or SP600125 + Ro31-8220, then subcellular localization of CRTC3 was examined. Bar = 100µm (**D**) Effect of JNK activation by anisomycin (ANS) on CREB/CRTC3 transcriptional activity. (**E**) Protein expression of melanogenic genes in Mel-Ab cells after 4 h of FSK or anisomycin + FSK treatment examined by western blotting. (**F**) Effect of JNK inhibition by SP600125 on phosphorylation and expression levels of CRTC2/3, CREB, AMPK, and JNK analyzed by western blotting.** (G)** CRTC3 phosphorylation in HEK-293T cells transfected with CRTC3 alone and CRTC3 with Flag tagged JNK1, JNK2, or JNK3 was compared by western blotting.

While exploring the signal pathway by which Ro31-8220 regulates CRTC activity, we found that Ro31-8220 dose-dependently increased JNK phosphorylation (Figure 4A and Figure S4A). To examine whether inhibition of CREB/CRTC activity by Ro31-8220 is due to enhanced JNK activity, we tested the effects of a specific JNK activator on CRTC3 phosphorylation and subcellular localization. Similar to Ro31-8220, the JNK agonist anisomycin increased the phosphorylation and cytosolic localization of CRTC3 and attenuated both FSK-stimulated dephosphorylation and nuclear migration of CRTC3 in Mel-Ab and B16F10 cells without altering CREB phosphorylation (Figure 4B, C and Figure S4B). Anisomycin also suppressed CREB transcriptional activity stimulated by CRTC3 overexpression or FSK exposure (Figure 4D) and attenuated cAMP-stimulated MITF upregulation in Mel-Ab cells (Figure 4E). Conversely, treatment with the JNK inhibitor SP6000125 stimulated the dephosphorylation and nuclear migration of CRTC3 and blocked Ro31-8220-induced phosphorylation and nuclear exclusion of CRTC3 (Figure 4C, F and Figure S4C). Moreover, the overexpression of JNK1/2/3 phosphorylates CRTC3, suggesting that JNK activation mediates Ro31-8220-induced phosphorylation and nuclear exclusion of CRTC (Figure 4G).

**Figure 5:**
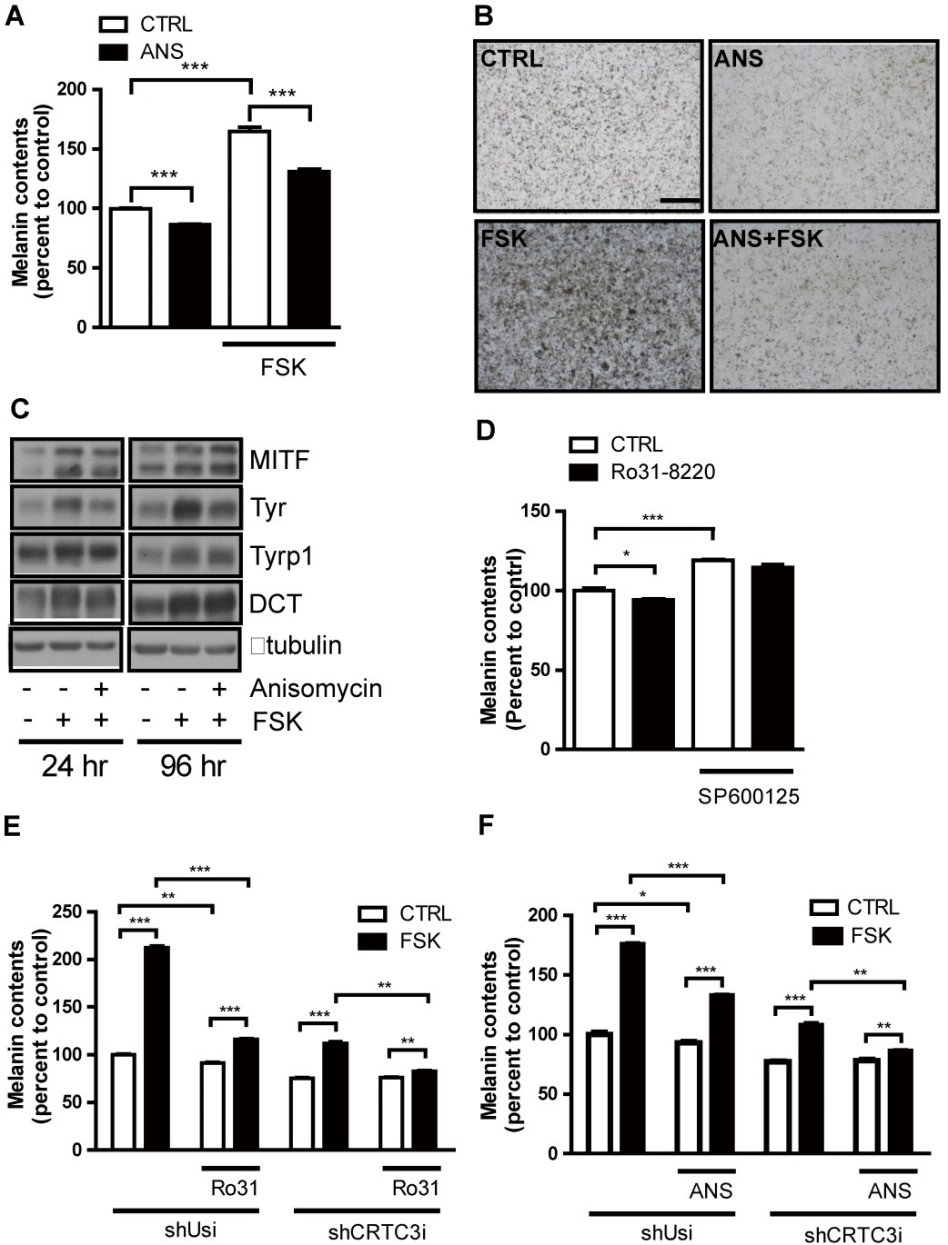
** JNK suppresses cAMP-stimulated melanogenesis in melanocytes.** (**A**-**C**) Mel-Ab cells were treated with anisomycin (ANS), FSK, or anisomycin + FSK for 96 h and examined for melanin content (**A**, **B**) and expression of melanogenic genes (**C**). Bars = 2000 µm in A and B. (**D**) Melanin content of Mel-Ab cells treated with SP600125, Ro31-8220, or Ro31-8220 + Sp600125 for 96 h. (**E**, **F**) Control (shUsi) or CRTC3 knockdown (shCRTC3i) Mel-Ab cells were treated with (**E**) Ro31-8220 (Ro31), FSK, or Ro31-8220 + FSK or (**F**) anisomycin (ANS), FSK, or anisomycin + FSK for 96 h and melanin content measured.

We next examined whether modulation of JNK signaling per se impacts on melanogenesis. Activation of JNK with anisomycin dose-dependently reduced melanin accumulation in Mel-Ab cells without affecting cell viability (Figure S5A-C). Moreover, anisomycin suppressed cAMP-stimulated tyrosinase expression and melanin production (Figure 5A-C). Conversely, the JNK inhibitor SP600125 dose- dependently increased melanin accumulation in Mel-Ab cells (Figure S6A-C) and mitigated suppression of melanin production by Ro31-8220 (Figure 5D). To confirm whether the anti-melanogenic activities of Ro31-8220 and anisomycin are dependent on the CRTC/CREB pathway, we tested the effects of Ro31-8220 and anisomycin in CRTC3 knockdown cells. While Ro31-8220 and anisomycin strongly suppressed basal- and cAMP-stimulated melanin accumulation in Mel-Ab cells expressing a control shUSi, these effects were markedly reduced in CRTC3 knockdown cells (Figure 5E, F and Figure S7).

### Ro31-8220 suppresses UVR-induced melanin production in human skin cultures

**Figure 6:**
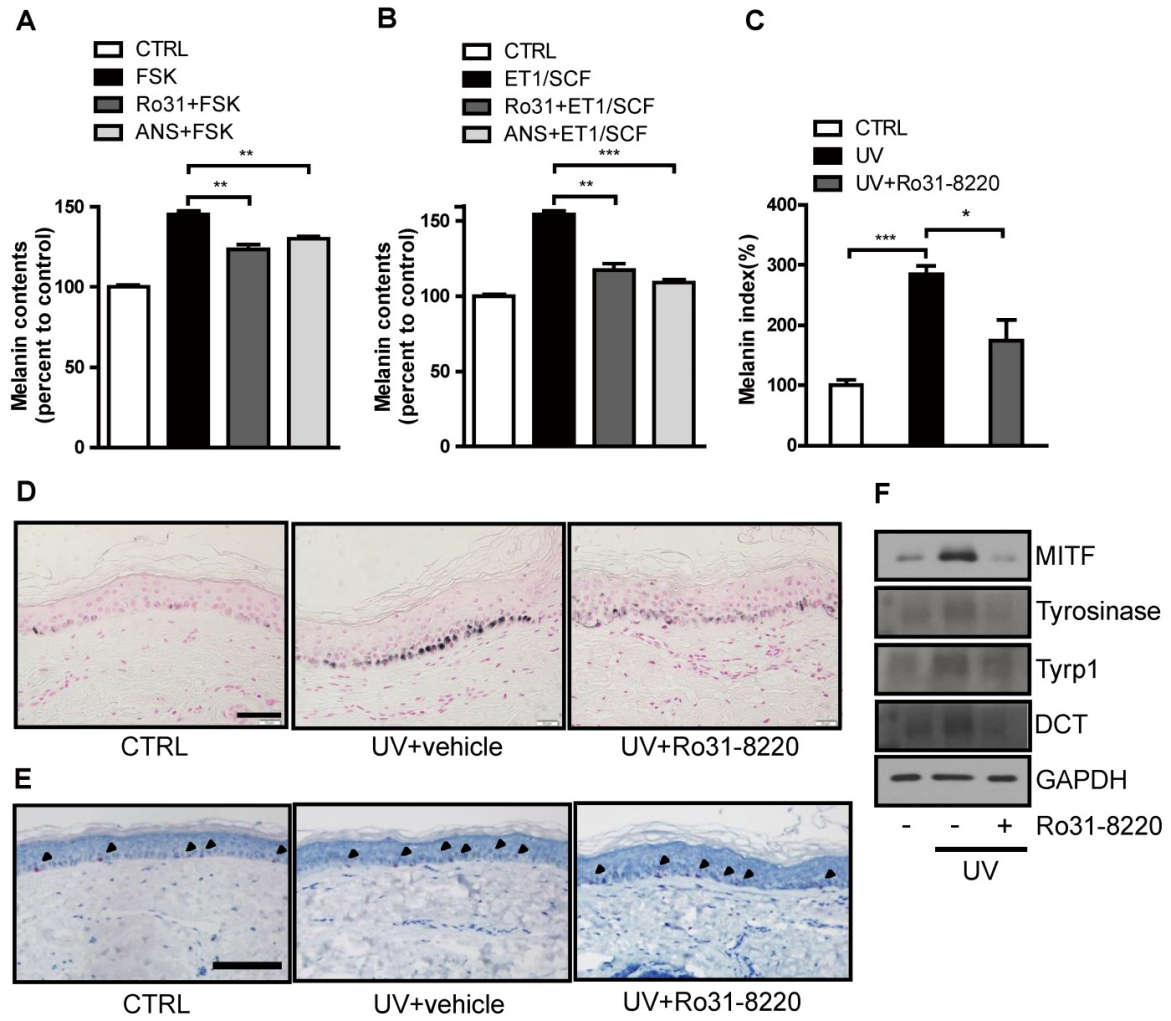
** Ro31-8220 suppresses UVR-induced melanin accumulation in melanocyte-keratinocytes cocultures and *ex vivo* human skin.** (**A**) Human primary melanocytes and keratinocytes in co-culture were treated with FSK or pretreated with Ro31-8220 or anisomycin (ANS) followed by FSK treatment. After 96 h, melanin content was examined. (**B**) Human primary melanocytes and keratinocytes co-culture were treated with ET-1/SCF or pretreated with Ro31-8220 or anisomycin (ANS) followed by ET-1/SCF. After 96 h of treatment, melanin content was examined. *Ex vivo* human skin tissues were exposed to UVB (150 mJ/cm^2^) or co-treated with Ro31-8220 plus UVB. After 96 h, skin tissues were harvested. (**C**) Quantification of melanin accumulation (melanin index). (**D**) Representative images of Fontana-Masson-stained paraffin-embedded sections treated with vehicle (CTRL), UVB + vehicle, or UVB + Ro31-8220. Bars = 100 µm. (**E**) Microscopic images of melan-A immunohistochemistry for melanocyte staining. Arrow heads indicate positive melan-A staining (Red color) Bar = 100 µm. (**F**) Protein expression of melanogenic genes in human skin tissue exposed to vehicle only (CTRL), UVR, or UVR+Ro31-8220 as assessed by western blotting.

We next examined whether Ro31-8220 also demonstrates anti-melanogenic activity in a human ketatinocyte-melanocyte co-culture system and *ex vivo* organotypic cultures of human skin. Both FSK and endothelin-1 (ET-1)/stem cell factor (SCF) treatment increased melanin production in melanocyte-keratinocyte cocultures, and pretreatment with Ro31-8220 or anisomycin attenuated this melanogenic response (Figure 6A, B and Figure S8). In addition, UVR-induced melanin accumulation in organotypic human skin explants was significantly attenuated by Ro31-8220 (Figure 6C, D), while hematoxylin and eosin staining together with immunostaining for melan-A, Sox10, Ki67, and p53 revealed no signs of skin tissue damage, melanocyte, or keratinocyte toxicity (Figure 6E, Figure S9, and Figure S10). Further, Ro31-8220 application suppressed UVR-induced upregulation of MITF and tyrosinase expression (Figure 6F).

## Discussion

Although there are other transcription factors that play a role in melanogenesis, MITF is the most heavily studied as a pigmentation response to UVR reaching agreement that CREB/MITF axis is the most critical pathway in UVR/αMSH-stimulated adaptive melanogenesis. In fact, except for MITF, the expression level of transcription factors β-catenin, Lef1, and PAX3 was not altered by cAMP stimulation (Figure S11). The current study was motivated by recent findings that CREB-mediated expression of MITF and melanogenesis require CRTC requirement to the CREB complex 3, and that chemical regulators of CRTCs may be useful for modulating MITF expression and melanin production.

### Anti-melanogenic mechanisms of Ro31-8220

In this study we demonstrate that Ro31-8220, a compound identified by CREB activity screening, possesses anti-melanogenic activity associated with reduced MITF expression, tyrosinase activity, and melanin accumulation. Previous studies have demonstrated phosphorylation-dependent regulation of tyrosinase catalytic activity by PKCβ 9, 13-15. However, in line with the selectivity of Ro31-8220 for PKCα 16-18, Ro31-8220 did not appear to directly affect tyrosinase activity, but rather decrease of tyrosinase activity by Ro31-8220 resulted from reduced tyrosinase expression due to downregulation of MITF.

Full activation of CREB target gene expression requires phosphorylation of CREB at Ser^133^ by PKA as well as the dephosphorylation and nuclear translocation of CRTCs 12, 19. Our study found that Ro31-8220-mediated suppression of MITF expression was independent of CREB phosphorylation but associated with inhibition of CRTC3 nuclear entry. In line with these, MITF expression was markedly induced in UV or cAMP-stimulated conditions and the inhibitory activity of Ro31-8220 under the basal conditions of melanogenesis was weaker than cAMP-stimulated conditions. In that point of view, because Ro31-8220 does not possess melanocytotoxic activity, clinical or *in vivo* studies may be worth pursuing to find out whether it also may have a lightening effect on UVR-protected skin or non-lesional skin near hyperpigmented patches. The AMPK signaling pathway is the best known inducer of phosphorylation-dependent CRTC activity 12, 20; however contrary to our expectations, Ro31-8220 did not alter AMPK expression or activity. Instead, JNK activity was enhanced dose-dependently by Ro31-8220, and like Ro31-8220, activation of JNK by a specific agonist or JNK overexpression promoted phosphorylation and cytoplasmic retention of CRTC, thereby inhibiting MITF expression and melanin production. Conversely, inhibition of JNK enhanced melanin production by stimulating dephosphorylation and nuclear migration of CRTC3. Of note, although the actions of JNK inhibition resembled those of FSK qualitatively (Figure 4F), JNK inhibition enhanced melanogenesis only moderately compared with FSK (Figure S6). This lower melanogenic activity may be explained by the dual requirement of CRTC dephosphorylation and CREB phosphorylation for full activation of CREB-mediated transcription, while JNK inhibition activates CRTC and promotes nuclear translocation but does not phosphorylate CREB (Figure 4F). There has been an expanding interest in the mechanisms regulating CRTC activity and to date only ubiquitination 21, 22 and direct phosphorylation by AMPK/SIK had been confirmed as upstream regulators 12, 20. Our unexpected discovery that JNK directly phosphorylates CRTC2/3 to inhibit CREB-mediated transcription of MITF and melanin production is possibly the most important finding of this study. Interestingly, JNK activity also appeared to be interfered with cAMP signaling suggesting cross-talk between cAMP-PKA and JNK signaling pathways 23-26.

### Is CRTC a suitable target for pigmentation disorder treatment?

There are studies supporting the potential utility of targeting CRTC/CREB signaling for treatment of hyperpigmentation disorders 27. Solar lentigo, PIH, and melasma, the most common UVR-associated hyperpigmentation disorders, are associated with increased MITF expression 5. Subcutaneous FSK (PKA activation) induced darker hair color in mice 28 while the inhibition of PKA by alpha-viniferin suppressed melanin production and improved melasma in human patients 6. Expression of adiponectin, known to inhibit CREB target gene expression in liver, is reduced in the lesional skin of melasma patients, and adiponectin treatment suppressed melanin accumulation via blockade of CRTC nuclear entry 3. Our data further support the attractive utility of targeting the CREB signaling pathway, especially CRTC activity, as it involves more proximal mechanism of melanogenesis than tyrosinase inhibition. Thus, targeting CRTC warrants consideration as an alternative strategy for treatment of pigmentary disorders. It is also of great importance to test whether Ro31-8220 application can be extended to metabolic syndrome treatment since adipose tissue CRTC3 contributes to energy metabolism and genetic ablation of CRTC3 in mice improves diabetes and obesity phenotypes 8, 29-32. Indeed, pigmentary skin disorders are frequently associated with metabolic syndromes 33.

### Does Ro31-8220 have therapeutic potential for hyperpigmentary disorders?

Beltman et al. (1996) reported that Ro31-8220 inhibited dual-specificity phosphatase 1 (DUSP1) and that inhibition of DUSP1 enhanced JNK1 activity 34, suggesting that DUSP signaling may also be involved in the regulation of CRTC activity via JNK. Currently, more than 10 DUSP isoforms with MAPK phosphatase activity has been described, with each isoform specific for different MAPK family members 35, 36. However, only one report has implicated DUSP signaling in pigmentation 37. We suggest that one potentially valuable future research strategy for safe and effective agents for hyperpigmentation disorder treatment is to identify melanocyte specific or enriched DUSP isoforms with selectivity for JNK and testing whether targeted regulation of these DUSP isoforms can modulate melanin accumulation through alteration of CRTC activity.

High anti-melanogenic activity as well as the safety, stability, and skin permeability in patients are the essential criteria for clinical applications for hyperpigmentation disorders 38. Regarding the bioavailability of topicals, their effective transepidermal delivery into human skin is challenging; thus, the development of liposome, microemulsions or nanocrystal delivery systems has been tested. Recently, diacetylcaffeic acid cyclohexyl ester (DACE) and rottlerin, small molecule drugs that inhibit CREB, have been shown to reduce melanin deposition in pig and mouse skin 7, 39. The present candidate, Ro31-8220 is also a small, soluble, and lipophilic drug, which likely has topical delivery efficiency. Indeed, in cultured melanocytes, Ro31-8220 reduced UVR- stimulated MITF expression and melanin deposition in human keratinocyte-melanocyte cocultures and *ex vivo* organotypic human skin without causing cellular damage. Nevertheless, further assessment of the topical bioavailability, biostability, and toxicity should be examined in human skin.

Although MITF regulation is the most critical step for effective treatment of pigmentary disorders, knowledge of MITF regulation and function is still very limited. Therefore, identifying CRTC3 and JNK as MITF regulators is an important advance in melanocyte biology 40. Deficiency of MITF may result in hypopigmentary disorders and vitiligo as well as more severe disease phenotypes such as Waardenburg syndrome, a disease associated with MITF mutation. Blockade of the JNK/CRTC pathway shown to regulate MITF in this study but may spare cAMP-dependent MITF expression since full activation requires both CREB and CRTC activities. Collectively, our findings suggest that targeted CREB and CREB/CRTC3 inhibitors such as Ro31-8220 or its analogs are potential test drugs for clinical trials.

## Supplementary Material

Supplementary figures and table.

## Figures and Tables

**Figure 7:**
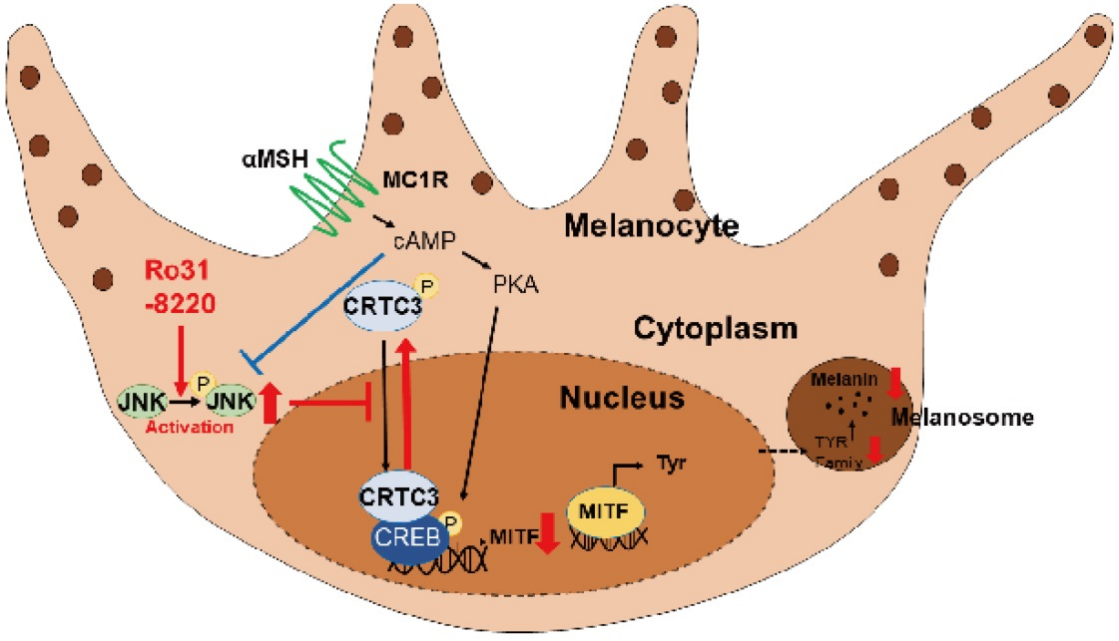
** Schematic model of melanogenic modulation by JNK-CRTC3/CREB-MITF signaling.** In response to UVR exposure, αMSH secreted from keratinocytes activates melanocortin receptor 1 (MC1R) on melanocytes, which in turn triggers cAMP generation, PKA activation, CREB phosphorylation, and nuclear entry of CRTC3, which promotes MITF expression and induction of downstream melanogenic genes. Activation of JNK by RO31-8220 or anisomycin directly phosphorylates CRTC3, thereby inhibiting nuclear translocation and suppressing UV-induced MITF expression and melanogenesis.

## Abbreviations

CRE: cAMP-responsive elements
CREB: CRE-binding protein
CRTC: CREB-regulated transcription coactivator
DCT: dopachrome tautomerase
FSK: forskolin
MEFs: mouse embryonic fibroblasts
MITF: microphthalmia-associated transcription factor
Rot: rottlerin
Tyr: tyrosinase
TYRP-1: tyrosinase-related protein-1
