# Supplementary material for: JNK suppresses melanogenesis by interfering with CREB-regulated transcription coactivator 3-dependent MITF expression
Source: Theranostics. 2020 Mar 4;10(9):4017–29. doi: 10.7150/thno.41502 (PMC7086364; doi:10.7150/thno.41502)
Supplement: Supplementary file 1 — Supplementary figures and table. [file thnov10p4017s1.pdf]

Supplementary Materials

Supplementary figures

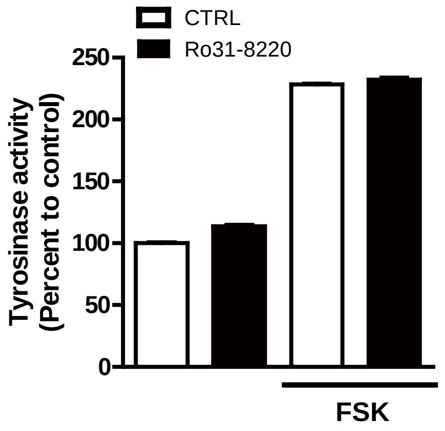

Figure S1. Short-term effects of Ro31-8220 on tyrosinase activity in Mel-Ab cells. Vehicle treated cells and cells treated with FSK for 72 h were then treated with vehicle or Ro31-8220 for 2 h, followed by tyrosine activity assays.

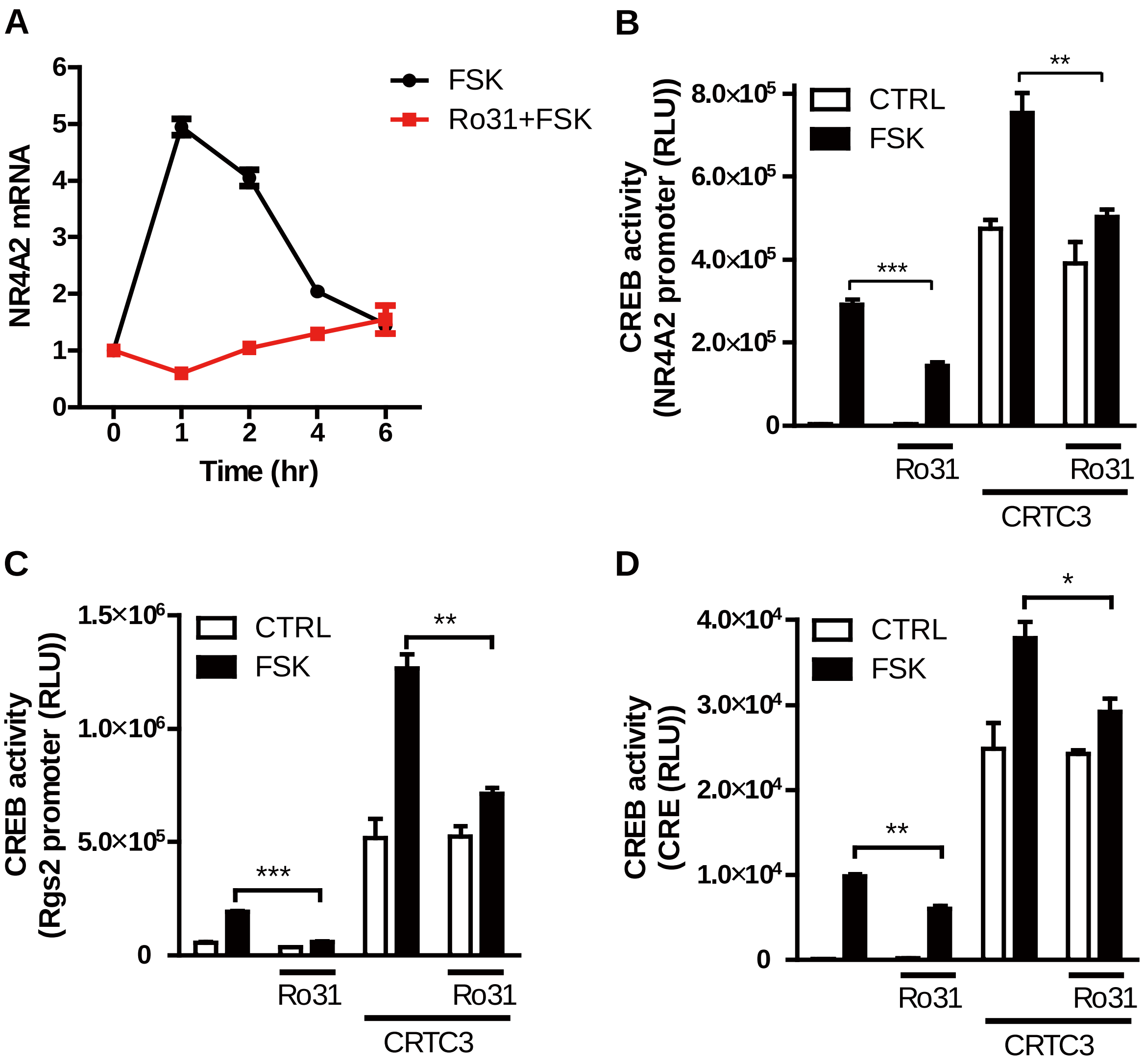

Figure S2. Effects of Ro31-8220 on CREB and CRTC3 transcriptional activity assessed by (**A**) measuring mRNA expression of the representative CREB target gene NR4A2 and assessing **(B)** NR4A2, **(C)** Rgs2 promoter activity, and **(D)** synthetic CRE (CGATGTCA) derived luciferase activity.

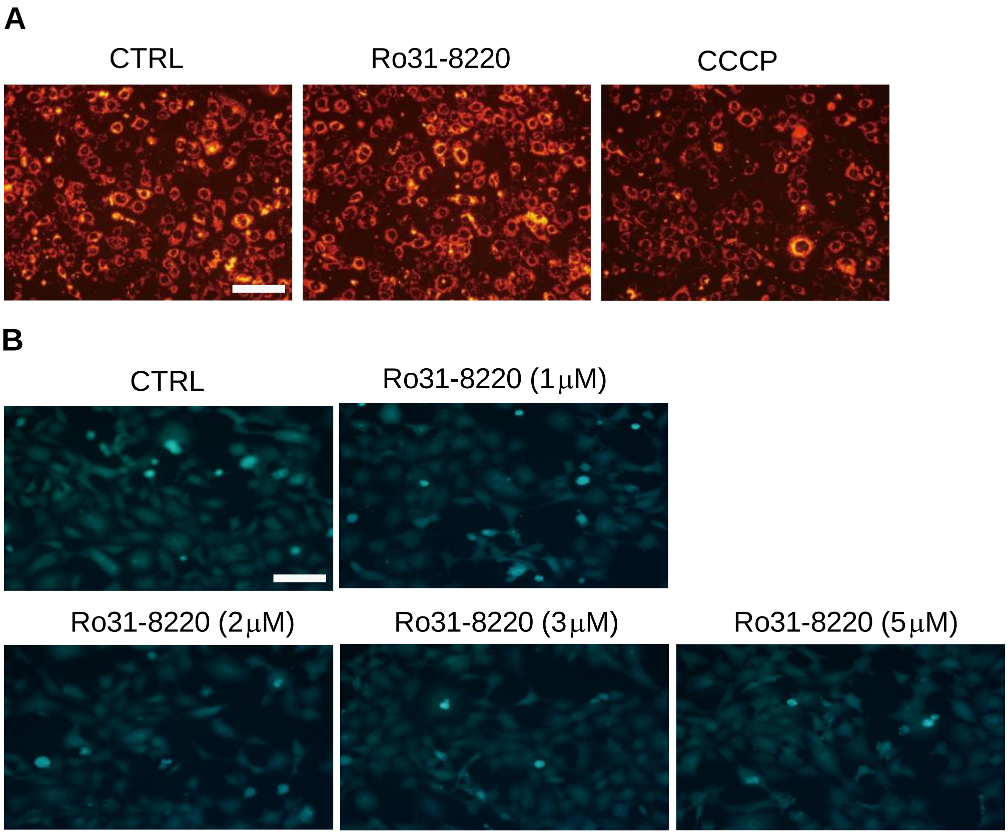

Figure S3. The effects of Ro31-8220 on (**A**) mitochondrial membrane potential and (**B**) intracellular Ca^{2+} levels that regulate AMPK activity via ATP production and CamKK respectively were examined by fluorometry using JC-1 and Fluo-4 staining. CCCP was used for mitochondrial membrane depolarization. Bars = 500 μm

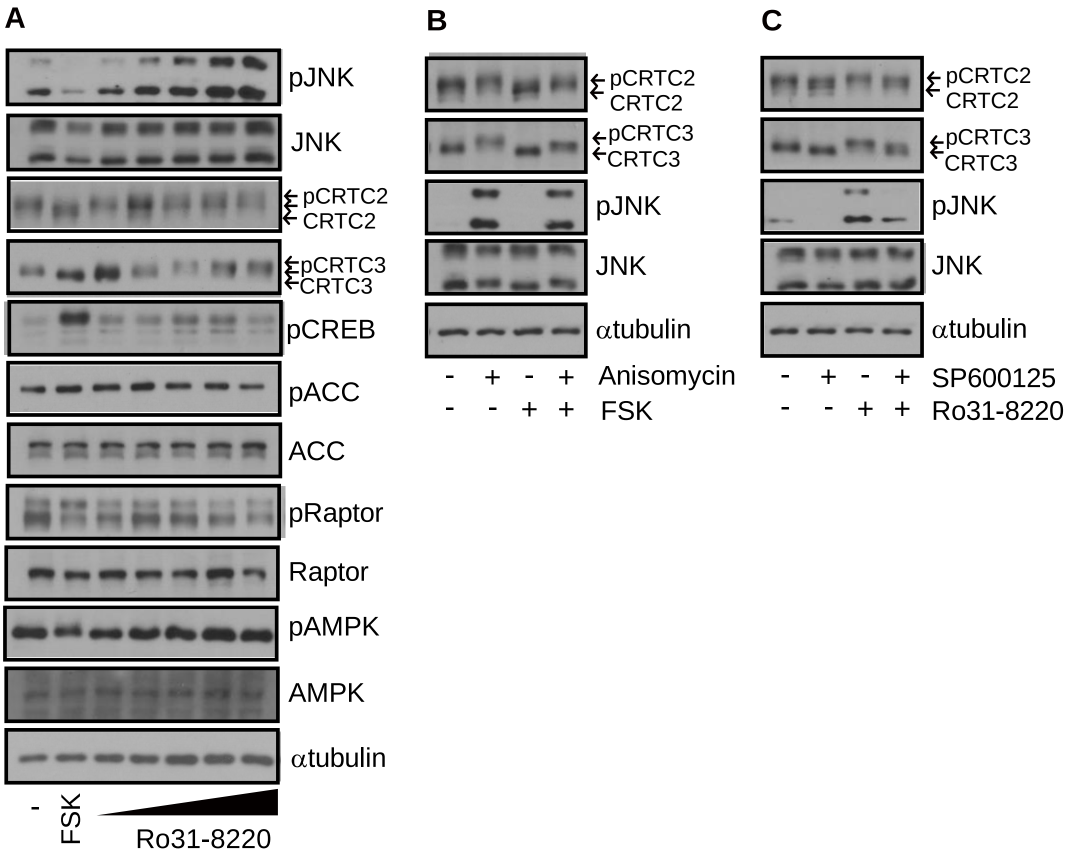

Figure S4. Short-term effects of forskolin, Ro31-8220, and anisomycin on expression and phosphorylation of JNK and CRTC/CREB pathway proteins. (**A**) Mouse embryonic fibroblasts (MEFs) were treated with 10 µM FSK or 0.01–2 µM Ro31-8220 for 1 h and the expression and phosphorylation of CRTC2/3, CREB, AMPK, Raptor, ACC, and JNK examined by western blotting. (**B**) MEFs were treated with anisomycin, FSK, or pretreated with anisomycin for 1 h then FSK for 1 h, and the phosphorylation and expression of CRTC2/3 and JNK were analyzed by western blotting. (**C**) Effects of JNK inhibition by SP600125 on phosphorylation and the expression of CRTC2/3 and JNK were analyzed using western blotting.

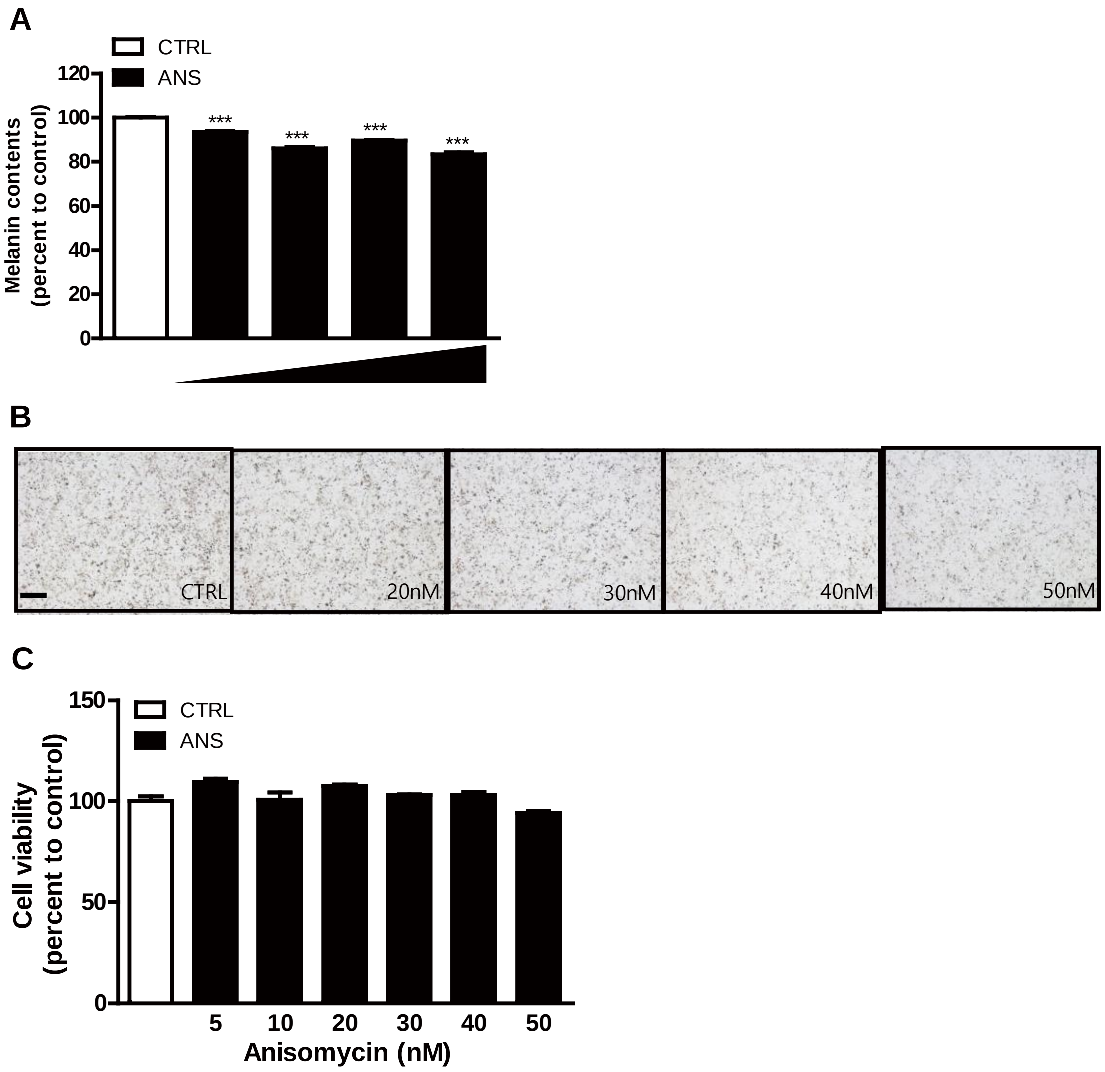

Figure S5. Effects of anisomycin on Mel-Ab cell melanin content and viability. (**A**, **B**) Mel-Ab cells were treated with 20–50 nM anisomycin for 96 h followed by (**A**) measurement of melanin content and (**B**) microscopic image acquisition. Bars: 2000 μm. (**C**) Mel-Ab cell viability after treatment with 5–50 nM anisomycin treatment for 96 h as measured by 3-(4,5-dimethylthiazol-2-yl)-2,5-diphenyltetrazolium bromide (MTT) assay. CTRL: vehicle, ANS: anisomycin.

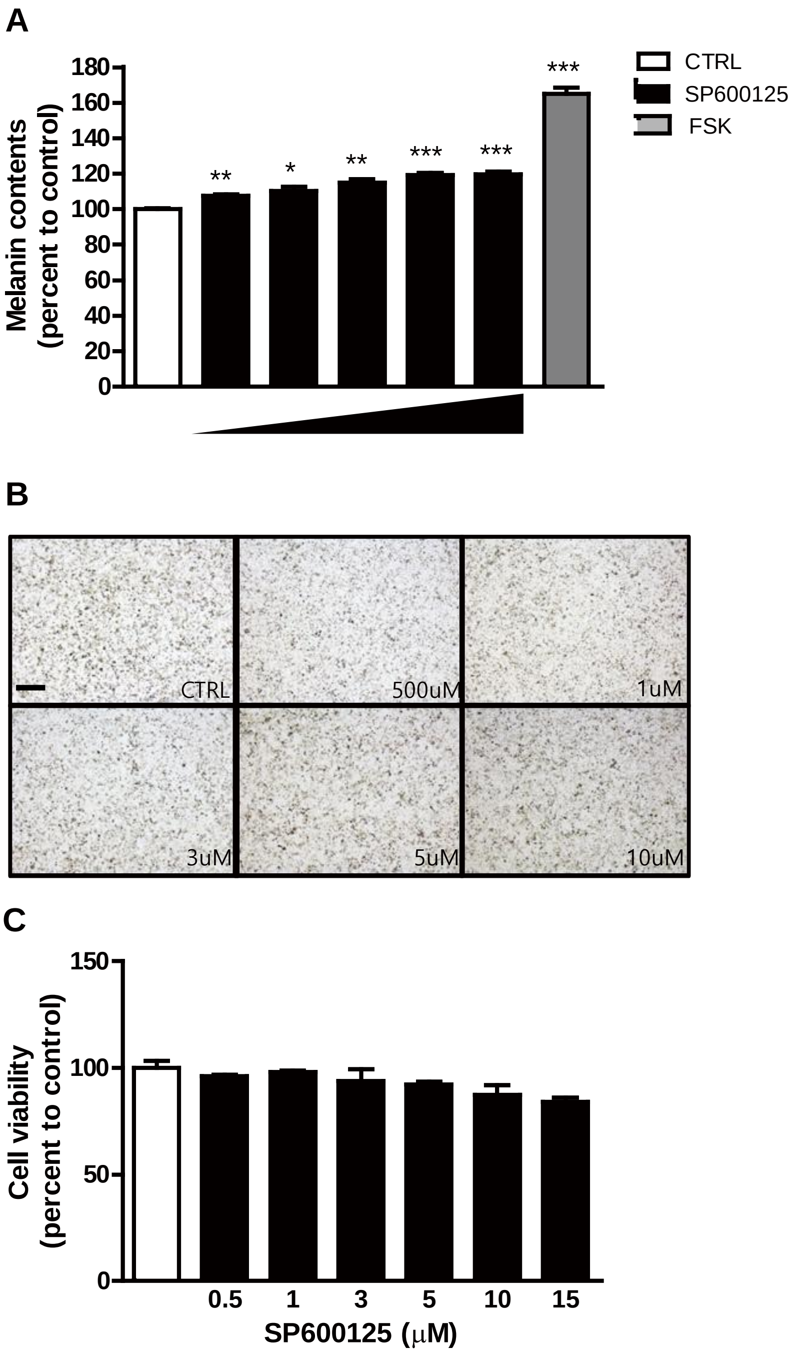

Figure S6. Effects of FSK and SP600125 on Mel-Ab cell melanin content and cell viability. (**A**, **B**) Mel-Ab cells were treated with 10-μM FSK or 0.5–15 μM SP600125 for 96 h, followed by (**A**) measurement of melanin content and (**B**) microscopic image acquisition. Bars: 2000 μm. (**C**) Mel-Ab cell viability following 0.5–15-μM SP600125 treatment for 96 h as measured by MTT assay. CTRL: vehicle treatment

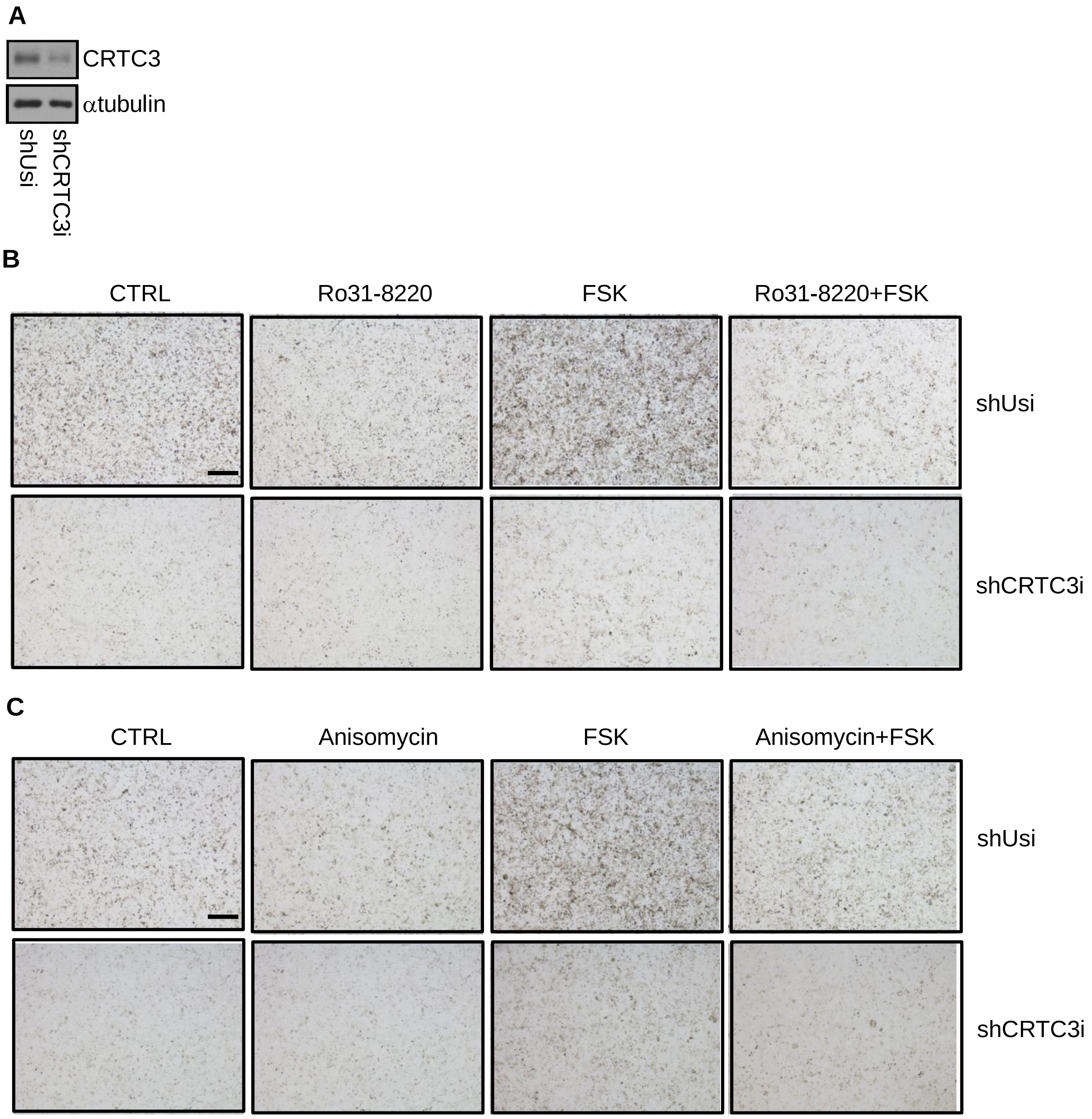

Figure S7. Effects of Ro31-8220 under CRTC3 knockdown. (**A**) Mel-Ab cells were infected with lentivirus encoding shUsi (control) or shCRTC3i (CRTC3 knockdown). CRTC3 knockdown efficiency was examined by comparing expression levels by western blotting. (**B**) Microscopic images of control (shUsi) and CRTC3 knockdown (shCRTC3i) Mel-Ab cells after 96 h of exposure to vehicle, Ro31-8220, FSK, or Ro31-8220 + FSK. Bars: 2000 μm. (**C**) Microscopic images of control (shUsi) and CRTC3 knockdown (shCRTC3i) Mel-Ab cells after 96 h of exposure to vehicle, anisomycin, FSK, or anisomycin + FSK Bars: 2000 μm

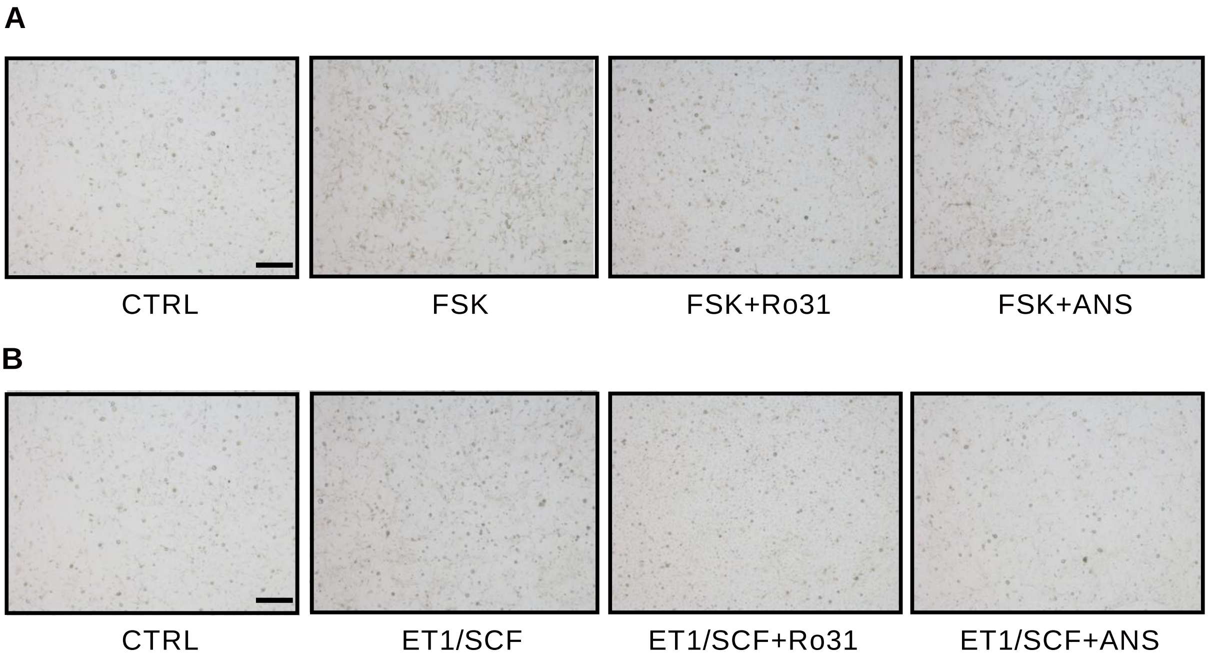

Figure S8. Effects of Ro31-8220 on melanin content in human primary keratinocyte–melanocyte cocultures. (**A**) Human primary melanocytes and keratinocytes in co-culture were treated with FSK or FSK plus either Ro31-8220 (Ro31) or anisomycin for 96 h and melanin content compared. (**B**) Human primary melanocytes and keratinocytes in co-culture were treated with ET-1/SCF or ET-1/SCF plus either Ro31-8220 (Ro31) or anisomycin for 96 h and melanin content compared. Bars: 2000 μm

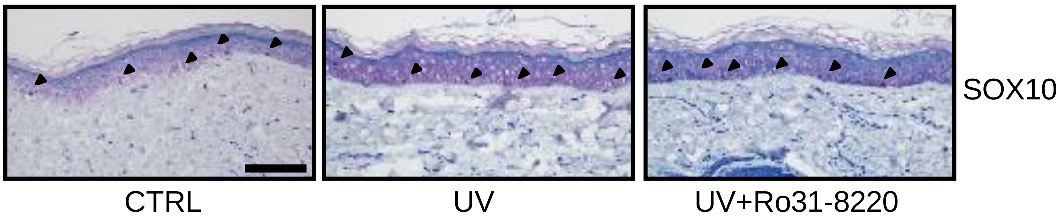

Figure S9. SOX10 immunostaining of organotypic human skin cultures exposed to vehicle (CTRL), UVR, or UVR + Ro31-8220. Arrow heads indicate SOX10 immunopositive cells. Bars: 100 μm

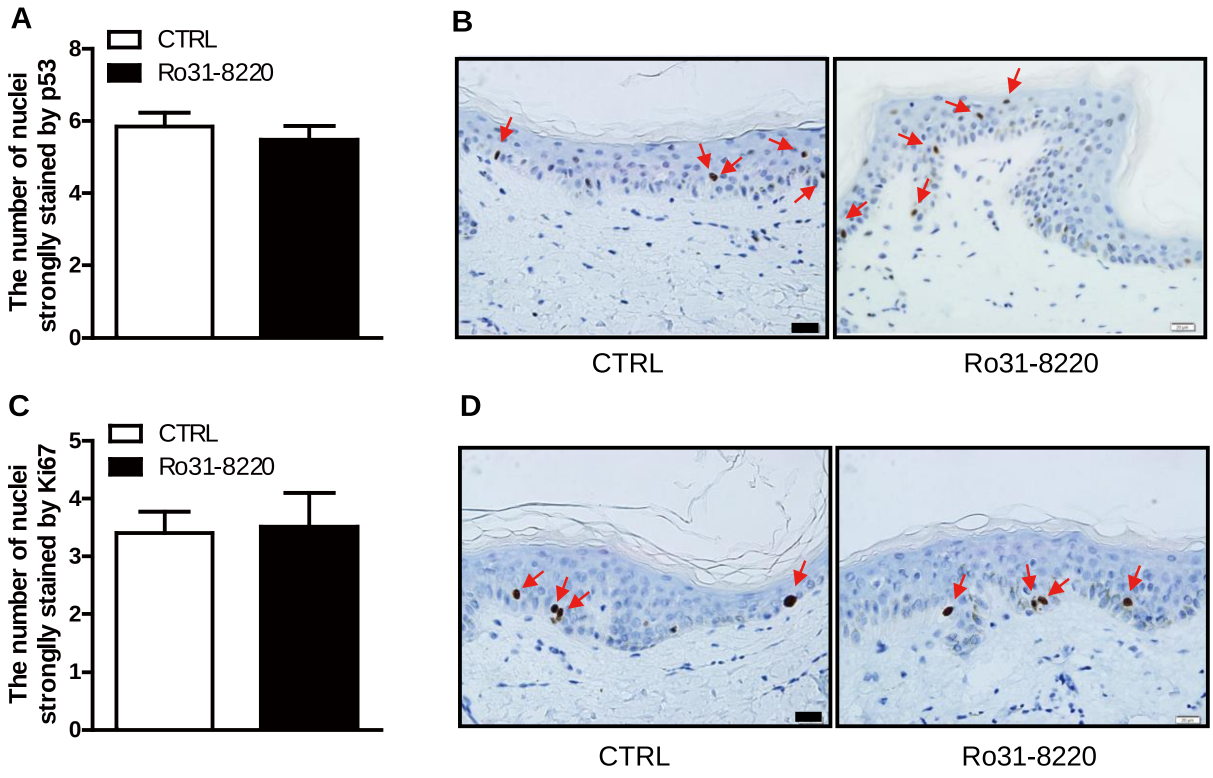

Figure S10. Quantification and representative microscopic images of immunoreactive cells in vehicle- or Ro31-8220-treated *ex vivo* human skin based on nuclear expression of **(A,B)** p53 and **(C,D)** Ki-67 by immunohistochemistry: Data represent mean numbers of positive cells ± s.e.m from serial 25 consecutive sections. Arrows indicate p53 or Ki67 immunopositive cells. Bars: 20 μm

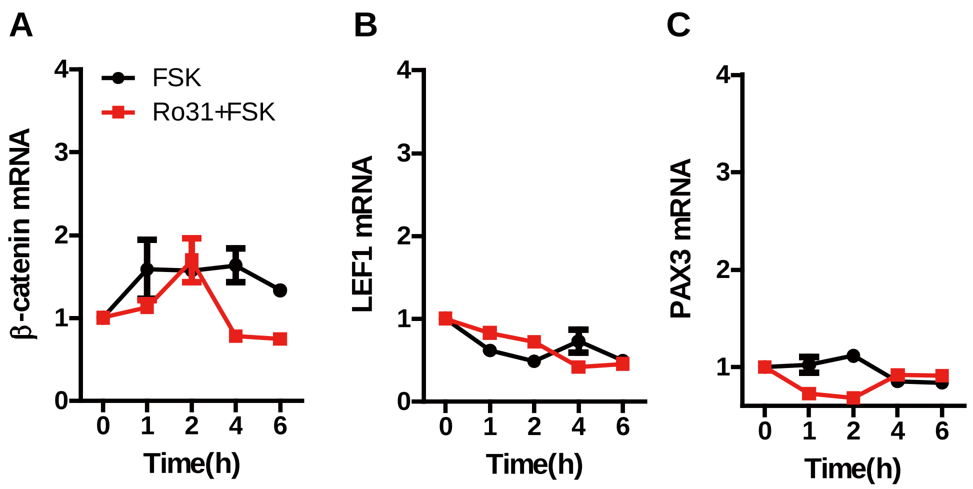

Figure S11. Short-term effects of FSK, and Ro31-8220 (Ro31) + FSK treatment (1–6 h) on expression levels of (A) β-catenin, (B) LEF1, and (C) PAX3

Supplementary table

Table S1. Primer sets for qRT-PCR

Gene	Forward primer	Reverse primer
L32	5'-AGATCCTGATGCCCAACATC-3'	5'-CAGCTCCTTGACATTGTGGA-3'
NR4A2	5'-AGTCTGATCAGTGCCCTCGT-3'	5'-GATCTCCATAGAGCCGGTCA-3'
MITF	5'-TGAAGCAAGAGCATTGGCTA-3'	5'-TCCACAGAGGCCTTGAGAAT-3'
TYR	5'-TTATGCGATGGAACACCTGA-3'	5'-ACTGGCAAATCCTTCCAGTG-3'
TYRP1	5'-TCACTGATGCGGTCTTTGAC-3'	5'-CTGACCTGGCCATTGAACTT-3'
DCT	5'-TCCTAACCGCAGAGCAACTT-3'	5'-TCTCCATTAAGGGCGCATAG-3'
